# Hyperspectral multi-trait modeling for rapid assessment of rice photosynthetic light-response traits under field conditions

**DOI:** 10.3389/fpls.2026.1968739

**Published:** 2026-10-13

**Authors:** Xinfeng Yao, Xiuguo Zheng, Huifeng Sun, Sheng Zhou, Wei Wang, Linyi Li

**Affiliations:** 1 Institute of Agricultural Science and Technology Information, Shanghai Academy of Agricultural Sciences, Shanghai, China; 2 Key Laboratory of Intelligent Agricultural Technology (Yangtze River Delta), Ministry of Agriculture and Rural Affairs, Shanghai, China; 3 Eco-environmental Protection Research Institute, Shanghai Academy of Agricultural Sciences, Shanghai, China; 4 Shanghai Engineering Research Centre of Low-carbon Agriculture (SERCLA), Shanghai, China; 5 Key Laboratory of Low-carbon Green Agriculture in Southeastern China, Ministry of Agriculture and Rural Affairs, Shanghai, China; 6 Shanghai Spark-X Technology Co., Ltd., Shanghai, China

**Keywords:** hyperspectral sensing, multi-trait prediction, photosynthetic traits, precision agriculture, rice

## Abstract

Light-response-curve (LRC) derived photosynthetic phenotyping traits, including the maximum net photosynthetic rate (PNmax), light-saturation point (Isat), light-compensation point (Icomp), and dark respiration rate (Rd), are important indicators of photosynthetic performance. However, conventional LI-6400-based measurements require tens of minutes per leaf, severely limiting high-throughput phenotyping and field-based stress monitoring. Here, we developed a hyperspectral approach that simultaneously estimates four key LRC-derived photosynthetic traits (PNmax, Isat, Icomp, and Rd) from leaf hyperspectral data. The framework was evaluated using observations collected under field conditions encompassing three years, three rice varieties, four nitrogen levels, three irrigation treatments, three leaf positions, and multiple measurement dates. Partial Least Squares Regression (PLSR) and machine learning models—including Backpropagation Artificial Neural Network (BP-ANN), Convolutional Neural Networks (CNN), and Support Vector Regression (SVR)—were tested with full spectrum and variable selection methods (Competitive Adaptive Reweighted Sampling, CARS; Successive Projections Algorithm, SPA; and Uninformative Variable Elimination, UVE). Variable selection generally improved model performance, with CARS providing the most stable overall results across the four traits. Multi-trait (MT) models outperformed single-trait (ST) counterparts, particularly on the held-out test set. Among all methods, nonlinear models achieved higher accuracy for individual traits. PLSR2+CARS provided the most consistent overall predictive performance across the four traits, achieving test-set R^2^ values of 0.84, 0.69, 0.52, and 0.59 and RMSE values of 2.53 μmol (CO_2_) m^−2^ s^−1^, 344.08 μmol (photon) m^−2^ s^−1^, 11.48 μmol (photon) m^−2^ s^−1^, and 0.58 μmol (CO_2_) m^−2^ s^−1^ for PNmax, Isat, Icomp, and Rd, respectively. These results indicate that leaf hyperspectral sensing combined with MT modeling has potential for high-throughput assessment of rice photosynthetic performance at the leaf level under field conditions.

## 1 Introduction

Plants undergo photosynthetic acclimation in response to environmental conditions, involving changes in the photosynthetic apparatus associated with variations in incoming solar irradiance, temperature, water availability, nutrient availability, and CO_2_ concentration (Kull, 2002; Murchie et al., 2002; Luo and Keenan, 2020). Light-response curves (LRCs) at the leaf level describe the relationship between photosynthesis and light intensity. Typically, the net photosynthetic rate increases approximately linearly under low irradiance and then gradually approaches saturation as irradiance increases (Ye, 2007). From these curves, a set of physiologically meaningful parameters can be derived. The maximum net photosynthetic rate (PNmax) represents light-saturated carbon-assimilation capacity; the light-saturation point (Isat) indicates the irradiance required to approach the maximum net assimilation rate; the dark respiration rate (Rd) quantifies respiratory carbon loss in darkness; and the light-compensation point (Icomp) represents the irradiance at which photosynthetic CO_2_ uptake balances respiratory CO_2_ release. Together, these parameters characterize leaf-level photosynthetic phenotypes and their responses to changing environmental conditions and are widely used in breeding selection and crop stress diagnosis (Araus et al., 2018; Silva-Perez et al., 2018). However, acquiring a complete LRC with a portable gas-exchange system (e.g., LI-6400) typically requires approximately 60 minutes per leaf (Yao et al., 2025a), which prevents large-scale phenotyping of breeding panels or stress-screening trials in the field.

Vegetation indices (VIs) based on specific wavelength combinations in the visible and near-infrared (VNIR) region have been widely explored for estimating diverse plant structural and biochemical indicators, such as biomass and green leaf area, chlorophyll, and nitrogen, as well as photosynthetic performance (Thenkabail et al., 2000). In the framework of the light-use efficiency (LUE) model, variation in photosynthetic carbon assimilation is determined by the amount of absorbed photosynthetically active radiation (APAR) and the efficiency (LUE) with which this absorbed energy is converted into fixed carbon (Monteith and Moss, 1977). The normalized difference vegetation index (NDVI) has been widely used as an indicator of vegetation greenness, biomass, and green leaf area and is closely related to the fraction of absorbed photosynthetically active radiation (fAPAR) (Myneni and Williams, 1994). Assessing LUE has been more challenging than measuring fAPAR from remote sensing, in part because LUE is highly variable in time and space (Gamon et al., 2016). The photochemical reflectance index (PRI), associated with xanthophyll-cycle activity, provides a means of tracking short-term changes in photosynthetic LUE (Gamon et al., 1992; Peñuelas et al., 2011). Over longer time scales, chlorophyll/carotenoid-sensitive reflectance index (CCI) has also been shown to track seasonal changes in photosynthetic rates (Gamon et al., 2016). The red-edge chlorophyll index (CI_red-edge_) is sensitive to variation in leaf chlorophyll content (Gitelson et al., 2003). These established indices demonstrate that specific spectral regions are physiologically associated with photosynthetic function: the green region, particularly around 531 nm, is sensitive to changes related to the xanthophyll cycle; the red region (650–680 nm) is strongly influenced by total chlorophyll content and the size of the light-harvesting antenna; the red-edge region (700–730 nm) is particularly sensitive to chlorophyll content, and the NIR spectral region is influenced primarily by internal leaf structure and multiple scattering (Jacquemoud and Baret, 1990; Ollinger, 2011; Gamon et al., 2016). Established VIs have demonstrated that predefined combinations of a small number of VNIR bands can provide useful proxies for vegetation biochemical and structural traits, but have generally shown only moderate predictive accuracy for some photosynthetic traits (Fu et al., 2020; Lu et al., 2025).

More recently, leaf-level hyperspectral spectroscopy combined with machine learning has been widely explored for estimating photosynthetic phenotyping traits, such as net photosynthetic rate (PN), maximum carboxylation rate (Vcmax), and maximum electron transport rate (Jmax) (Ainsworth et al., 2014; Yendrek et al., 2017; Silva-Perez et al., 2018; Meacham-Hensold et al., 2019; Kumagai et al., 2022; Zhang et al., 2023; Guo et al., 2026). Hyperspectral approaches have also been applied to predict LRC-derived traits, including PNmax, Isat, Rd, and Icomp (Yao et al., 2025a). Compared with predefined VIs based on a limited number of wavelengths, hyperspectral modeling can exploit information distributed across the continuous spectrum and identify spectral regions associated with target physiological traits. These studies, typically based on Partial Least Squares Regression (PLSR) or machine learning techniques, highlight the promise of hyperspectral sensing for non-destructive plant photosynthetic phenotyping. Nevertheless, most existing models focus on single traits (ST) and are not suitable for accounting for the intrinsic interdependence among photosynthetic phenotyping traits, which may limit prediction accuracy and biological interpretability. To address this limitation, a multi-trait (MT) model enables the simultaneous prediction of multiple traits with a single model by leveraging shared features and statistical dependencies among traits, thereby potentially improving prediction accuracy and reducing the need for separate models for each trait (Zhai et al., 2025). In plant science, MT models have also shown clear advantages: MT models improved above-ground biomass estimation in tropical forests (Huy et al., 2024); MT regression outperformed ST approaches by up to 10% for predicting element concentrations in tomato leaves (Aguilar-Ariza et al., 2024); and hyperspectral imaging combined with MT models has enabled simultaneous estimation of chlorophyll, water content, and multiple physicochemical indices in crops like rice (Zhai et al., 2025) and tea (Jiang et al., 2025). Collectively, these advances highlight the promise of MT models for capturing trait interdependencies, though their application to jointly estimating multiple LRC-derived photosynthetic traits from a single hyperspectral scan remains under-explored.

Hyperspectral reflectance data typically contain a large number of wavelengths, making them high-dimensional and often strongly collinear (Wold et al., 2001; Heckmann et al., 2017; Burnett et al., 2021), which can obscure key spectral-physiological relationships. Therefore, identifying informative feature wavelengths is essential to reduce dimensionality, enhance model interpretability, and improve the accuracy and robustness of photosynthetic trait estimation. A number of studies have combined hyperspectral techniques with variable selection to identify key wavelengths for estimating plant biochemical traits, improving model efficiency and reliability (Sonobe et al., 2021). Variable selection aims to identify a small set of representative wavelengths, reducing redundant information and generating more concise and informative spectral data. Common approaches include Competitive Adaptive Reweighted Sampling (CARS) (Li et al., 2009), Successive Projections Algorithm (SPA) (Araújo et al., 2001; Shen et al., 2020), and Uninformative Variable Elimination (UVE) (Sonobe et al., 2021). These methods have been applied to estimate various plant traits, such as moisture content (Ji et al., 2020; Song et al., 2023), fatty acid content (Song et al., 2023), photosynthetic pigments (Shen et al., 2020), and stress indicators (Shen et al., 2020). However, the traditional variable selection methods are typically trait-specific and not designed for the simultaneous MT estimation pursued here.

To address these challenges, this study develops a hyperspectral framework for the simultaneous estimation of multiple photosynthetic phenotyping traits in rice under field conditions. Building on the same dataset used in our previous single-response PLSR study (Yao et al., 2025a), the present study shifts the focus from separate prediction of individual LRC traits to their simultaneous estimation and shared wavelength selection within a unified MT framework. Specifically, this study aimed to: (i) extend SPA, UVE, and CARS from single-response variable selection to a multi-response formulation in which wavelength importance is evaluated across PNmax, Isat, Icomp, and Rd; (ii) identify a unified wavelength subset that retains predictive information shared among the four traits; (iii) compare full-spectrum and wavelength-selected models under ST and MT formulations using PLSR, BP-ANN, CNN, and SVR; and (iv) evaluate whether the MT framework provides more consistent predictive performance on the held-out test set across observations collected under heterogeneous field conditions, encompassing different years, rice varieties, nitrogen levels, irrigation treatments, leaf positions, and measurement dates. This framework has the potential to reduce per-leaf light-response acquisition from approximately 60 minutes with a portable gas-exchange system (e.g., LI-6400) to a single hyperspectral scan, providing a basis for high-throughput leaf-level photosynthetic phenotyping.

## 2 Materials and methods

The overall workflow of this study is illustrated in **Figure 1**. The framework integrates field experiments, leaf hyperspectral reflectance and LRC measurements, spectral preprocessing and derivation of LRC traits, data partitioning, wavelength selection, ST and MT modeling, and model evaluation using a held-out test set. Leaf hyperspectral reflectance and LRCs were collected across different rice varieties, nitrogen and irrigation treatments, leaf positions, years, and measurement dates. After spectral preprocessing and derivation of PNmax, Isat, Icomp, and Rd, full-spectrum and selected-wavelength inputs were evaluated using ST and MT implementations of PLSR, BP-ANN, CNN, and SVR. Model performance was evaluated on the held-out test set using R^2^ and RMSE.

**Figure 1:**
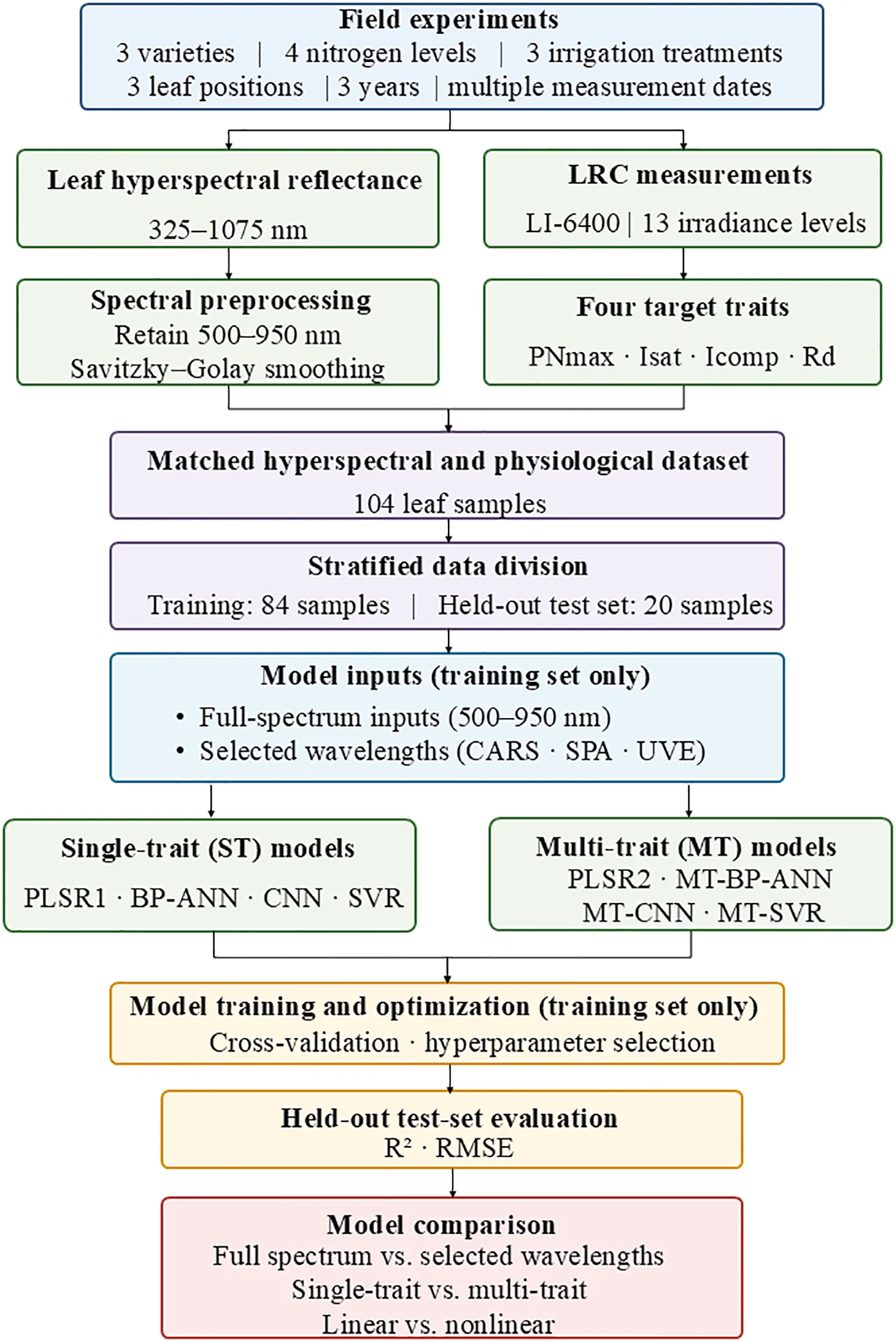
Workflow of the proposed hyperspectral framework for estimating rice photosynthetic phenotyping traits.

### 2.1 Experimental design

The photosynthetic phenotyping traits of rice under field conditions were obtained from field experiments conducted during 2015–2017 at the Low-Carbon Agricultural Engineering Technology Research Center, Zhuanghang Comprehensive Experimental Station of the Shanghai Academy of Agricultural Sciences, Shanghai, China (30°53′N, 121°23′E). The experiments involved both nitrogen fertilizer and irrigation treatments, with three replications for each treatment. Each experimental plot covered an area of 56 m^2^ (7 m × 8 m), and standard agronomic practices for conventional high-yield rice production systems were applied. Details of the experimental design are summarized in **Table 1**.

**Table 1:** Summary of rice field experiments: (a) nitrogen fertilizer experiment (2015–2017) and (b) irrigation experiment (2017).

(a) Nitrogen fertilizer experiment (2015–2017)					
Year	Treatment	Variety	Measurement dates	Leaf position	Sample number
2015	N0, N1, N2, N3	Huayou14	2015-09-09, 2015-10-02	L1, L2, L3	19
2016	N0, N1, N2, N3	Huayou14	2016-09-12, 2016-09-20, 2016-10-24, 2016-11-03	L1, L2, L3	30
2017	N0, N1, N2, N3	Huhan61	2017-08-23, 2017-08-28, 2017-09-13, 2017-10-07	L1, L2, L3	43
(b) Irrigation experiment (2017)					
Year	Treatment	Variety	Measurement date	Leaf position	Sample number
2017	HH100, HH50, HH20	Huhan61	2017-09-12	L1, L2	6
2017	XS100, XS50, XS20	Xiushui134	2017-09-12	L1, L2	6

The nitrogen fertilizer experiment was carried out from 2015 to 2017, with four nitrogen application levels: 0, 100, 200, and 300 kg hm^−2^ (denoted as N0, N1, N2, and N3, respectively). Rice varieties used were Huayou14 in 2015 and 2016, and Huhan61 in 2017. Nitrogen fertilizer was applied in three stages: 50% as basal, 30% as tillering, and 20% as heading fertilizer. In addition, phosphorus (P_2_O_5_) at 100 kg hm^−2^ was applied as basal fertilizer, and potassium (K_2_O) at 225 kg hm^−2^ was applied in two splits (44% basal and 56% heading).

The irrigation experiment was conducted in 2017, with three irrigation levels corresponding to 100%, 50%, and 20% of the irrigation amount used under conventional irrigation management. The 100% treatment followed the conventional irrigation practice used to meet the water requirements of rice, while the 50% and 20% treatments received 50% and 20%, respectively, of the irrigation amount applied to the 100% treatment at each irrigation event. Two rice varieties were used: Huhan61 (HH100, HH50, HH20) and Xiushui134 (XS100, XS50, XS20). All irrigation treatments received nitrogen fertilizer at the N2 level (200 kg hm^−2^), together with the same phosphorus and potassium fertilization scheme as in the nitrogen experiment.

### 2.2 Leaf hyperspectral reflectance measurements and pre-processing

Leaf hyperspectral reflectance was collected in the field using a non-imaging point spectroradiometer (ASD HandHeld2; 325–1075 nm, approximately 3 nm spectral resolution; Analytical Spectral Devices Inc., Boulder, CO, USA). The instrument was equipped with a fiber optic plant probe and a leaf clip containing an internal calibrated light source, which ensured stable illumination during measurement. To reduce systematic error, calibration was performed with a standard white reference panel, while a black panel was used to minimize stray light interference. For subsequent analysis, the spectral range was restricted to 500–950 nm, resulting in 451 wavelength variables at 1-nm sampling intervals.

Spectral measurements were taken on the adaxial side of the same leaves used for gas exchange assessment. Three leaves corresponding to the flag leaf (L1) and the two successive lower leaves (L2 and L3) were sampled from each plot. For each leaf, three different positions were selected, and five consecutive scans were acquired per position. A white reference measurement was recorded before scanning each leaf. In total, fifteen spectra per leaf were collected and subsequently averaged to generate a single representative reflectance spectrum.

Because of strong sensor noise at both spectral ends, only the range between 500 and 950 nm was retained for subsequent modeling. Prior to analysis, spectra were smoothed using the Savitzky–Golay (SG) filter to reduce high-frequency noise while preserving the original spectral features.

### 2.3 Photosynthetic phenotyping traits

LRCs of rice leaves were recorded in the field using a portable photosynthetic gas exchange system (LI-6400, LI-COR, Inc., Lincoln, Nebraska, USA) equipped with a red-blue LED light source. Measurements followed an automated program with irradiance levels of 2000, 1600, 1300, 1000, 800, 600, 400, 200, 150, 100, 50, 25, and 0 µmol (photon) m^−2^ s^−1^. The reference CO_2_ concentration was maintained at 400 ppm, and leaf temperature was allowed to follow ambient conditions. Leaf widths were measured to adjust gas exchange rates to the actual leaf area. Data collection was performed between 10:00 a.m. and 4:00 p.m. on clear, calm days, shortly after the flag leaf had fully emerged, spanning late August to early November. In total, 104 LRCs were obtained, encompassing three years, three rice varieties, four nitrogen levels, three irrigation treatments, multiple leaf positions, and different measurement dates (leaf ages), as detailed in **Table 1**.

Four LRC-derived photosynthetic traits were considered in this study. The measured net PN–irradiance data were fitted using the Ye model (Equation 1) (Ye, 2007):

(1) $$PN=\phi_{\left(I_{0}-I_{comp}\right)}\times \frac{1-\beta \times I}{1+\gamma \times I}\times \left(I-I_{comp}\right)$$

where PN is the net photosynthetic rate (μmol (CO_2_) m^−2^ s^−1^), I is the photosynthetic photon flux density (μmol (photon) m^−2^ s^−1^), ϕ_(I0-Icomp)_ is the quantum yield with I between I_0_ and I_comp_ (μmol (CO_2_) μmol (photon)^−1^), I_comp_ is the light-compensation point (μmol (photon) m^−2^ s^−1^), and β and γ are the photoinhibition and saturation coefficients, respectively. Model fitting was performed using nonlinear least-squares regression in MATLAB R2016a (MathWorks, Natick, MA, USA), as described previously (Yao et al., 2025b).

PNmax and Isat were derived from the fitted model parameters according to the definitions of the Ye model. Isat was defined as the irradiance level beyond which a further increase in light intensity no longer resulted in an appreciable increase in PN, and PNmax was the net photosynthetic rate at Isat. These two parameters are regarded as representative indicators of photosynthetic activity under light-saturated conditions (Yao et al., 2025b). Icomp was estimated from a linear regression of PN against irradiance using the measurements at 0, 25, and 50 µmol (photon) m^−2^ s^−1^ (Ye, 2007) and was defined as the x-intercept (PN = 0) of the fitted regression. Rd was obtained directly from the LRC measurement at an irradiance of 0 µmol (photon) m^−2^ s^−1^ (Rd = −PN at zero irradiance).

### 2.4 Variable selection

In this study, three representative variable selection methods—CARS, SPA, and UVE—were employed to extract feature wavelengths from hyperspectral data. All variable-selection procedures were performed exclusively using the 84 observations in the training set, without using any observations from the held-out test set. Moreover, to identify common feature wavelengths across multiple photosynthetic phenotyping traits, an MT selection strategy was applied for each method.

For PLSR models, variable selection was directly integrated into model construction. For BP-ANN, CNN, and SVR models, the input wavelengths were taken from the best-performing subset selected by each of the three PLSR-based variable selection methods (CARS, SPA, and UVE) and were further reduced using PCA. In addition, the impact of variable selection on model performance was evaluated for both PLSR and machine learning models by comparing results with and without feature reduction.

#### 2.4.1 Competitive adaptive reweighted sampling

The CARS algorithm, originally proposed by Li et al (Li et al., 2009), is a wavelength selection method that iteratively eliminates uninformative variables by assessing their importance via the absolute values of regression coefficients in a PLSR model. The procedure begins with Monte Carlo sampling to randomly select a fixed proportion of calibration observations. In each iteration, an exponentially decreasing function is applied to remove variables with small regression coefficients, followed by adaptive reweighted sampling to select informative variables based on their weight values. This process is repeated multiple times, generating a series of variable subsets, and the optimal subset is determined by the minimum Root Mean Square Error (RMSE) of cross-validation from the PLSR model (Li et al., 2009).

In this study, we extended the conventional CARS method to enable simultaneous variable selection for multiple photosynthetic phenotyping traits. Instead of constructing separate PLSR models for each photosynthetic phenotyping trait, a PLSR2 (MT-PLSR) model was established, where the output matrix contained all target traits. The variable importance was assessed by calculating the mean of the absolute values of regression coefficients across all traits, thereby capturing the common spectral features that contribute collectively to the prediction of multiple traits.

#### 2.4.2 Successive projection algorithm

The SPA is a forward-selection method that identifies a subset of variables with minimal collinearity by projecting each candidate variable onto the orthogonal subspace of previously selected variables and selecting the one with the largest projection (Araújo et al., 2001).

In this study, SPA was extended to handle multiple output variables simultaneously. Instead of using a single response, a PLSR2 model was constructed at each iteration, and the projection values of candidate variables were weighted by the combined residuals across all outputs. This allows the selected subset to capture spectral features relevant to all photosynthetic phenotyping traits, rather than optimizing for a single trait at a time.

#### 2.4.3 Uninformative variable elimination

UVE is a wavelength selection algorithm based on PLSR coefficients that identifies informative wavelengths by comparing the stability of each variable to artificial noise, improving model predictive power. For ST-UVE, variable importance is evaluated for each single response using the mean-to-standard-deviation ratio of regression coefficients with thresholds derived from the maximum noise.

In this study, UVE was extended to handle multiple output variables simultaneously. A PLSR2 model was constructed, and the importance of each candidate variable was determined by averaging the absolute regression weights across all traits. Variables exceeding the corresponding thresholds were retained as the final optimized feature set.

### 2.5 Model development

#### 2.5.1 Data splitting strategy

A stratified random sampling strategy was applied to divide the dataset into a training set and a held-out test set. The dataset consisted of 104 leaf-level observations obtained from 104 different leaves, each containing paired leaf hyperspectral and LRC measurements. The 104 observations used in the present study were the same as those reported in our previous study (Yao et al., 2025a). Whereas the previous study focused on separate single-response PLSR modeling, the present study uses this dataset to investigate MT modeling and shared wavelength selection for the simultaneous estimation of multiple LRC traits.

The data partition was performed at the leaf-observation level. For each trait, the data range was divided into five equal-width bins. The test set comprised 20 observations, aiming for four observations from each bin for every trait to ensure that the distribution of the four traits in the test set was representative of the overall dataset. The remaining 84 observations were used as the training set. Each leaf contributed only one paired hyperspectral–LRC observation to the dataset. Detailed information on the training/test assignment and experimental characteristics of all 104 observations is provided in **Supplementary Table S1**.

To achieve this, an iterative random search was conducted to identify 20 test observations that minimized deviations from the target distribution (four observations per bin). The search was repeated up to 10,000 iterations, and the subset with the smallest deviation was retained as the test set. Although perfect matching was not always possible, this stratified design ensured that the test set adequately covered the full range of variation for all four traits, thereby reducing potential bias in model evaluation (**Figure 2**). The held-out test set was excluded from variable selection, dimensionality reduction, hyperparameter optimization, and model fitting, and was used only for final model evaluation.

**Figure 2:**
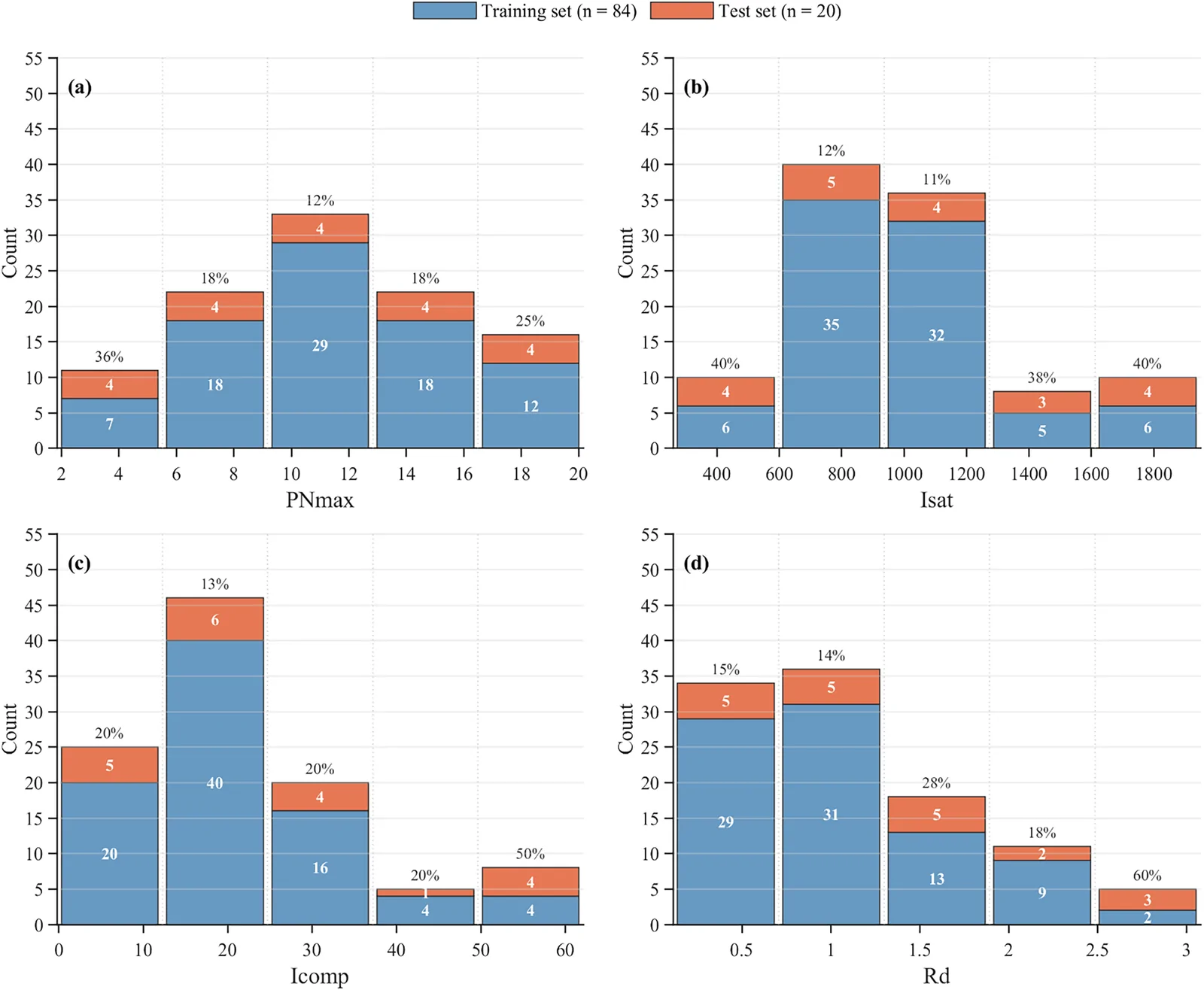
Count distribution of four photosynthetic phenotyping traits—PNmax **(A)**, Isat **(B)**, Icomp **(C)**, and Rd **(D)**—for the training (blue) and the test set (red).

For BP-ANN, CNN, and SVR model development, the training set was further randomly split into a training subset and a validation subset. The optimal hyperparameters were determined via five-fold cross-validation on the training subset, with mean squared error (MSE) as the selection criterion. During cross-validation, PCA was fitted using only the training folds and subsequently applied to the corresponding validation fold. The validation subset was then used for final verification of the selected hyperparameter configuration. After hyperparameter selection and validation, PCA was refitted using the entire training set. The model was retrained using the entire training set (training + validation subsets), and the fitted PCA transformation was applied to the held-out test set for final model evaluation.

#### 2.5.2 Partial least squares regression

PLSR is a widely used multivariate statistical technique that can be implemented in two modes: PLSR1, which models a single response variable, and PLSR2, which simultaneously models multiple correlated response variables. In this study, PLSR2 was applied to jointly predict multiple photosynthetic phenotyping traits (Y) from hyperspectral reflectance data (X) while separate PLSR1 models were developed for each trait for comparison.

In PLSR2, the predictor matrix X is projected onto a set of common latent variables (LVs) that are optimized to maximize the covariance between X and the response matrix Y. This approach offers three key advantages: (1) simplified model development and interpretation; (2) improved predictive performance when response variables are strongly correlated; and (3) simultaneous graphical interpretation of multiple responses. In this study, the optimal number of LVs was determined using repeated double cross-validation (rdCV) on the training dataset to minimize the risk of overfitting and ensure robust predictive performance (Filzmoser et al., 2009; Yao et al., 2025a).

#### 2.5.3 Backpropagation artificial neural network

The BP-ANN model was used to capture the nonlinear relationships between hyperspectral features and multiple photosynthetic phenotyping traits. The BP-ANN model consisted of an input layer, one or two hidden layers, and an output layer, with sigmoid activation functions in the hidden layers and a linear activation function in the output layer. The number of output neurons corresponded to the number of target variables being predicted. Both single-trait BP-ANN models (ST-BP-ANN) and multi-trait BP-ANN models (MT-BP-ANN) were developed to evaluate prediction performance under different modeling strategies.

To enhance model generalization and prevent overfitting, a systematic grid search strategy was applied to optimize key hyperparameters. Specifically, the candidate training algorithms included the Bayesian regularization backpropagation (trainbr), scaled conjugate gradient (trainscg), Levenberg–Marquardt (trainlm), and resilient backpropagation (trainrp). The hidden layer configurations were tested across multiple structures, including single-layer with 8 or 12 neurons, and double-layer with (20, 10), (16, 8), (24, 12), (32, 16), and (10, 5) neurons. Additionally, the regularization coefficient was tuned over a range of values (0.0, 0.01, 0.05, 0.1, 0.2) to balance the trade-off between model complexity and overfitting risk.

#### 2.5.4 Convolutional neural network

The one-dimensional CNN was developed to capture localized spectral features and model the nonlinear relationships between hyperspectral reflectance and multiple photosynthetic phenotyping traits. The CNN architecture consisted of a sequence input layer, two convolutional blocks, a global average pooling (GAP) layer, and an output layer. Each convolutional block included a convolutional layer with kernel size k and f filters (increased to 2f in the second block), followed by batch normalization, a rectified linear unit (ReLU) activation function, and a dropout layer with rate dr. The GAP layer was used to aggregate spectral features into compact representations, which were then passed to a fully connected layer with a number of neurons equal to the number of target traits, and finally to a regression layer with MSE loss. Both single-trait CNN (ST-CNN) and multi-trait CNN (MT-CNN) models were implemented to evaluate their predictive performance under different modeling strategies.

To enhance model generalization and prevent overfitting, a grid search strategy was employed to optimize key hyperparameters. Specifically, the candidate parameter space included kernel sizes (3 and 5), numbers of filters (16 and 32), dropout rates (0, 0.2, and 0.5), learning rates (1×10^−3^ and 5×10^−4^), and batch sizes (8, 16, and 32). Model training was carried out using the Adam optimizer for up to 200 epochs, with early stopping applied when validation error did not improve for 20 consecutive checks.

#### 2.5.5 Support vector regression

Four photosynthetic phenotyping traits were also predicted from hyperspectral data using both single-trait SVR (ST-SVR) and multi-trait SVR (MT-SVR). The MT-SVR extends the conventional SVR framework to multiple outputs, allowing simultaneous estimation of all target variables while exploiting cross-output correlations among outputs. It employs a shared radial basis function (RBF) kernel and an ϵ-insensitive cost function, and is trained through an iterative reweighted least-squares procedure to maintain sparsity and robustness to noise and nonlinearities. Key hyperparameters—including the penalty parameter C (10^-3^–10^4^), kernel width (10^-3^–10^4^), and ϵ-insensitive threshold (10^-3^–1)—were optimized using a genetic algorithm. Hyperparameter selection for both ST-SVR and MT-SVR models was conducted via five-fold cross-validation on the training set to ensure robust generalization and reproducibility of the results.

### 2.6 Model validation metrics

Model accuracy was assessed using a held-out test set to ensure unbiased evaluation. For each predicted photosynthetic phenotyping trait, the coefficient of determination (R^2^) was used to quantify the proportion of variance in the observed values explained by the model, while the RMSE was calculated to measure the average magnitude of prediction errors in the same units as the response variable. Higher R^2^ values and lower RMSE values indicate better model performance.

## 3 Results

### 3.1 Correlation analysis of photosynthetic phenotyping traits

**Figure 3** presents a scatter plot matrix illustrating the correlations among photosynthetic phenotyping traits. Notably, PNmax showed a strong positive correlation with Isat (R = 0.86, p< 0.01), suggesting that leaves with higher light-saturated photosynthetic capacity tended to reach their maximum net photosynthetic rates at higher irradiance, potentially consistent with coordinated photosynthetic acclimation. However, the relationship between PNmax and Isat is not necessarily fixed, because both parameters can change with the light-acclimation state of leaves and other environmental and physiological conditions (Murchie et al., 2002; Luo and Keenan, 2020). Additionally, Icomp exhibited a significant positive correlation with Rd (R = 0.77, p< 0.01), which is physiologically plausible because a higher respiratory carbon loss in darkness generally requires greater photosynthetic carbon assimilation, and thus higher irradiance, to reach the light-compensation point (Kull, 2002). Isat also showed a moderate positive relationship with Rd (R = 0.30, p< 0.05). Compared with the close definitional relationship between Icomp and Rd, the association between Isat and Rd is more indirect and may reflect coordinated variation in overall metabolic strategy, chemical composition, and growth environment (Givnish, 1988).

**Figure 3:**
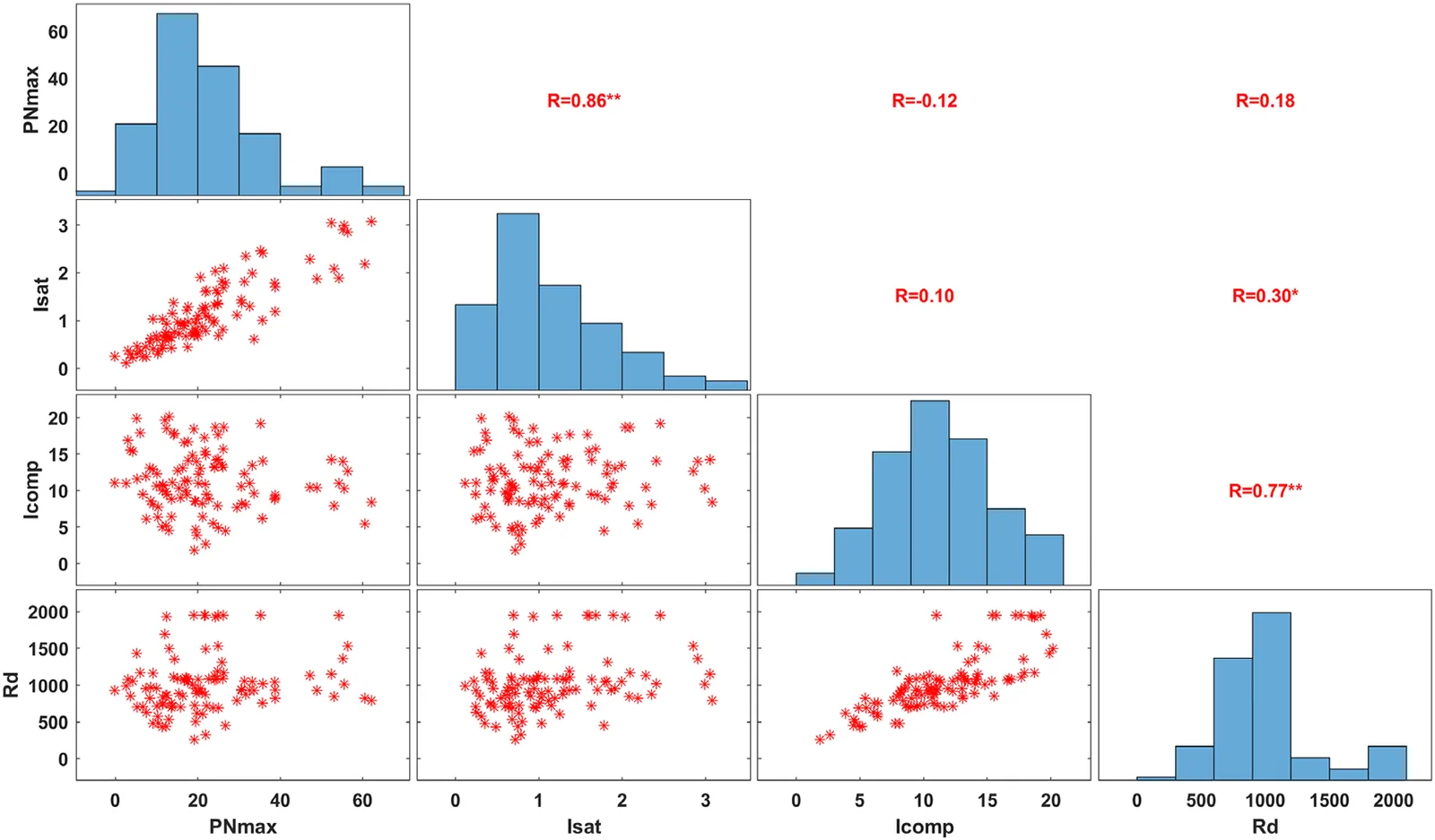
Correlations between measured photosynthetic phenotyping traits. The matrix of Pearson correlation coefficients between each pair of measured traits is shown above the diagonal, with the corresponding scatterplots displayed below the diagonal, while diagonal elements display histograms representing the distribution of each trait. Significance levels are indicated by asterisks (*p< 0.05, **p< 0.01).

### 3.2 Key wavelengths associated with rice photosynthetic traits

Three variable selection methods—CARS, SPA, and UVE—were applied to identify key wavelengths for the prediction of PNmax, Isat, Icomp, and Rd. For the PLSR2 model, specifically, the SPA method selected 71 wavelengths, which were broadly and unevenly distributed in the full spectrum. The UVE method produced 89 selected wavelengths, yielding the densest distribution among the three methods, with wavelengths concentrated in the green region (500–537 nm), the orange-to-red region (594–661 nm), the red-edge region (682–713 nm), and several NIR clusters (813–819, 872–902, and 920–950 nm). In contrast, the CARS method identified 35 wavelengths, forming a relatively compact set with representative wavelengths at 535, 552, 594, 612, 640, 674, 700, 720, 813, 882, and 934 nm. The wavelengths identified across the three methods were predominantly located in the green (533–537 nm), red (654–662 nm), red-edge (711–714 nm), and NIR regions (813–819 nm, 881–888 nm, 899–904 nm, and 920–935 nm) (**Figure 4**). The green wavelengths are close to spectral regions sensitive to pigment composition and xanthophyll-related changes; the red wavelengths coincide with strong chlorophyll absorption; and the red-edge wavelengths are highly sensitive to variation in chlorophyll status and related photosynthetic properties (Gamon et al., 2016). The NIR wavelengths are influenced primarily by internal leaf structure and multiple scattering, while the longer NIR wavelengths (920–935 nm) approach spectral regions sensitive to leaf water status (Jacquemoud and Baret, 1990; Ollinger, 2011). Thus, the wavelengths identified by the three variable-selection methods correspond to established VNIR spectral regions associated with plant pigments, photosynthetic function, leaf structure, and water status, providing physiological support for their relevance to photosynthetic phenotyping.

**Figure 4:**
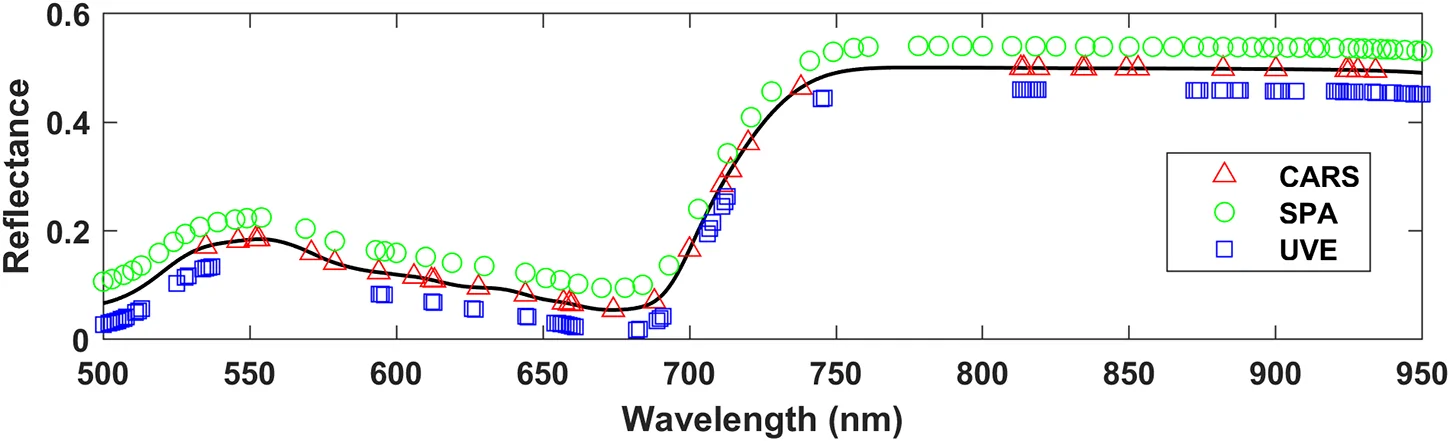
Wavelengths selected by three variable selection methods (CARS, SPA, and UVE) for the joint prediction of PNmax, Isat, Icomp, and Rd. The black line represents the mean reflectance spectrum of all observations, while the colored markers indicate the positions of the selected wavelengths.

In the PLSR1 setting, wavelengths selected by the three variable selection methods (CARS, SPA, and UVE) for individual photosynthetic phenotyping traits are shown in **Supplementary Figure S1**. Across the four traits, the three methods consistently emphasized the red-edge region (680–740 nm), along with additional selections in the green (500–550 nm) and near-infrared (760–860 nm) regions. While SPA tended to yield a larger and more continuous set of wavelengths, CARS and UVE identified more compact sets of discrete bands with strong predictive contributions. Despite differences in selection strategy, the three methods converged on key spectral regions essential for capturing both biochemical and structural information relevant to photosynthetic processes.

### 3.3 PLSR model performance

#### 3.3.1 Impact of variable selection on PLSR performance

To evaluate the impact of different variable selection strategies on model performance, CARS, SPA, UVE, and full-spectrum were compared under both PLSR1 and PLSR2 models. Overall, variable selection improved the predictive accuracy of PLSR1 and PLSR2 models relative to those constructed with the full-spectrum (**Table 2**). For instance, the test performance of Isat improved from R^2^ = 0.46 and 0.46, RMSE = 389.58 and 403.72 μmol (photon) m^−2^ s^−1^ with full-spectrum PLSR1 and PLSR2 models, respectively, to R^2^ = 0.50 to 0.69 and RMSE = 344.08 to 376.06 μmol (photon) m^−2^ s^−1^ after applying variable selection. Similar improvements in test were observed for Icomp and Rd: R^2^ increased from 0.51 and 0.53 for the full-spectrum PLSR1 and PLSR2 models, respectively, to a maximum R^2^ = 0.62 for Icomp, and from 0.47 and 0.49 to a maximum of 0.59 for Rd. In contrast, PNmax showed only modest gains in test (R^2^ = 0.76 to 0.84, RMSE = 2.47 to 2.72 μmol (CO_2_) m^−2^ s^−1^), as the full-spectrum PLSR1 and PLSR2 models already exhibited relatively strong performance (R^2^ = 0.76 and 0.81, respectively).

**Table 2:** PLSR1 and PLSR2 model performance for predicting photosynthetic phenotyping traits.

Model	Variable selection	Trait	LV	Model calibration		Held-out test	
				R^2^	RMSE	R^2^	RMSE
PLSR1	CARS	PNmax	10	0.79	1.76	**0.81**	**2.47**
		Isat	15	0.68	193.77	**0.54**	**364.42**
		Icomp	15	0.84	5.20	**0.51**	**11.15**
		Rd	11	0.69	0.36	0.44	0.64
	SPA	PNmax	15	0.83	1.58	0.76	2.71
		Isat	13	0.57	224.21	0.57	364.83
		Icomp	14	0.78	5.99	0.52	11.42
		Rd	14	0.73	0.33	**0.48**	**0.63**
	UVE	PNmax	15	0.83	1.58	0.76	2.72
		Isat	15	0.66	199.86	0.50	376.06
		Icomp	14	0.78	5.96	0.51	11.44
		Rd	14	0.73	0.33	0.47	0.63
	Full-spectrum	PNmax	15	0.83	1.58	0.76	2.71
		Isat	15	0.64	206.05	0.46	389.58
		Icomp	14	0.78	5.96	0.51	11.45
		Rd	14	0.73	0.33	0.47	0.63
PLSR2	CARS	PNmax	12	0.70	2.12	**0.84**	**2.53**
		Isat		0.46	251.74	**0.69**	**344.08**
		Icomp		0.66	7.52	0.52	11.48
		Rd		0.62	0.39	**0.59**	**0.58**
	SPA	PNmax	14	0.65	2.28	0.82	2.69
		Isat		0.40	264.57	0.58	375.86
		Icomp		0.68	7.28	**0.62**	**10.44**
		Rd		0.63	0.39	0.55	0.60
	UVE	PNmax	10	0.62	2.38	0.81	2.65
		Isat		0.35	276.31	0.61	369.81
		Icomp		0.64	7.69	0.57	10.89
		Rd		0.54	0.43	0.54	0.60
	Full-spectrum	PNmax	15	0.71	2.07	0.81	2.77
		Isat		0.48	247.49	0.46	403.72
		Icomp		0.72	6.83	0.53	11.44
		Rd		0.65	0.38	0.49	0.64

Bold values indicate the best performance on the held-out test set (highest R² and lowest RMSE) for each trait across variable-selection strategies, evaluated separately for PLSR1 and PLSR2.

For PNmax and Isat, both PLSR1 and PLSR2 models achieved their highest accuracies with CARS. Specifically, the PLSR1 and PLSR2 models reached test R^2^ = 0.81 and 0.84 (RMSE = 2.47 and 2.53 μmol (CO_2_) m^−2^ s^−1^) for PNmax, respectively, while they achieved test R^2^ = 0.54 and 0.69 (RMSE = 364.42 and 344.08 μmol (photon) m^−2^ s^−1^) for Isat, respectively. For Icomp, in PLSR1, CARS outperformed with test R^2^ = 0.51, RMSE = 11.15 μmol (photon) m^−2^ s^−1^, whereas in PLSR2, SPA provided superior performance with test R^2^ = 0.62, RMSE = 10.44 μmol (photon) m^−2^ s^−1^. For Rd, SPA yielded the best PLSR1 performance (R^2^ = 0.48, RMSE = 0.63 μmol (CO_2_) m^−2^ s^−1^ for test), while CARS led in PLSR2 models (R^2^ = 0.59, RMSE = 0.58 μmol (CO_2_) m^−2^ s^−1^ for test). Overall, these results demonstrate that variable selection can substantially enhance the robustness of PLSR models, especially for traits with low baseline accuracy (e.g., Isat and Rd). Among the three variable selection methods, CARS consistently delivered the most effective and stable performance, followed by SPA, while UVE showed the least improvement in this study.

#### 3.3.2 Comparison between PLSR1 and PLSR2

The performance of PLSR1 and PLSR2 models was further compared under the same variable selection settings. In general, PLSR1 achieved higher calibration accuracy for individual traits, particularly when combined with CARS or SPA. For example, PLSR1 with CARS achieved the best Isat calibration to R^2^ = 0.68, RMSE = 193.77 μmol (photon) m^−2^ s^−1^, and Icomp calibration to R^2^ = 0.84, RMSE = 5.20 μmol (photon) m^−2^ s^−1^. For PNmax and Rd, PLSR1 with SPA achieved the best calibration (R^2^ = 0.83, RMSE = 1.58 μmol (CO_2_) m^−2^ s^−1^, and R^2^ = 0.73, RMSE = 0.33 μmol (CO_2_) m^−2^ s^−1^). However, PLSR2 showed a clear advantage in simultaneously modeling correlated photosynthetic phenotyping traits, an approach that reduces the complexity of trait-specific modeling and often provides higher accuracy during test.

#### 3.3.3 Optimal PLSR model performance

Although PLSR1 models generally achieved the highest calibration accuracies for individual traits, PLSR2 combined with CARS proved to be the most effective strategy for MT prediction, particularly in test. Compared to PLSR1, PLSR2 combined with CARS delivered more consistent predictions across all traits, demonstrating its advantage in generalizing to unseen data. In this study, the optimal number of latent variables for the PLSR2 with CARS model was determined to be 12 using rdCV.

**Figure 5** illustrates the optimal predictions for all photosynthetic phenotyping traits across different nitrogen levels (N0–N3) and leaf positions (L1–L3), with the black 1:1 line indicating perfect agreement between observed and predicted values. Using PLSR2 with CARS, PNmax, Isat, Icomp, and Rd achieved calibration R^2^/RMSE of 0.70/2.12 μmol (CO_2_) m^−2^ s^−1^, 0.46/251.74 μmol (photon) m^−2^ s^−1^, 0.66/7.52 μmol (photon) m^−2^ s^−1^, and 0.62/0.39 μmol (CO_2_) m^−2^ s^−1^, respectively, with test R^2^/RMSE of 0.84/2.53 μmol (CO_2_) m^−2^ s^−1^, 0.69/344.08 μmol (photon) m^−2^ s^−1^, 0.52/11.48 μmol (photon) m^−2^ s^−1^, and 0.59/0.58 μmol (CO_2_) m^−2^ s^−1^, respectively. PLSR2 with CARS consistently outperformed the best PLSR1 results (**Supplementary Figure S2**), confirming its superior predictive capability.

**Figure 5:**
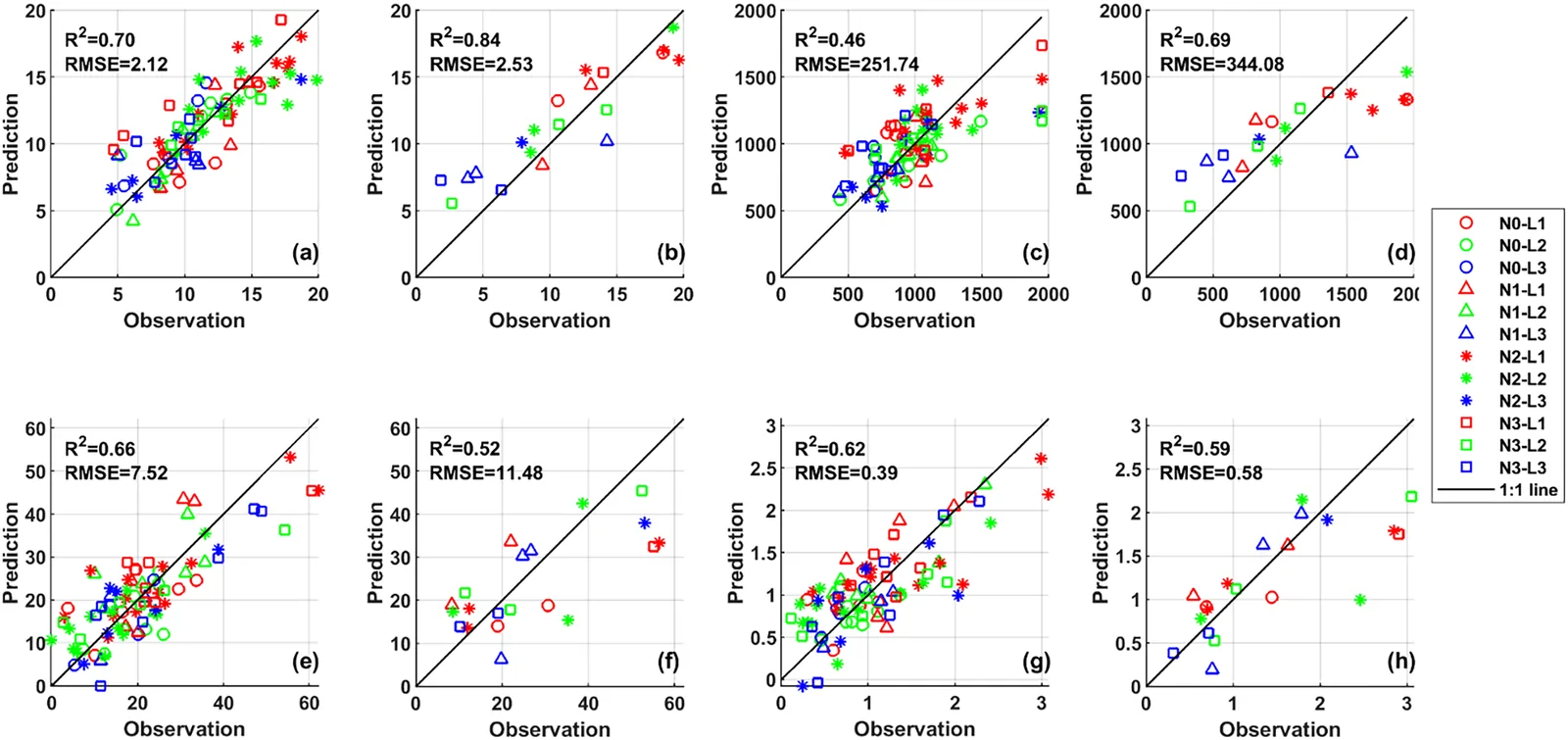
Scatter plots of observed versus predicted values for photosynthetic phenotyping traits (PNmax **(a, b)**, Isat **(c, d)**, Icomp **(e ,f)**, and Rd **(g, h)**) obtained using PLSR2 combined with CARS under different nitrogen levels (N0–N3) and leaf positions (L1–L3). Panels **(a, c, e ,g)** show the calibration results, whereas panels **(b ,d, f ,h)** show the test results. The black 1:1 line represents perfect agreement between observed and predicted values.

### 3.4 Machine learning model performance

Based on the PLSR model performance, CARS was identified as the most effective variable selection method. In subsequent analyses, CARS was combined with different machine learning models (BP-ANN, CNN, and SVR) to predict photosynthetic phenotyping traits. The model performance is summarized in **Table 3**. Prediction performance with full-spectrum was also evaluated (**Table 3**).

**Table 3:** Calibration and test performance of BP-ANN, CNN, and SVR models with full-spectrum and CARS variable selection for predicting photosynthetic phenotyping traits, comparing multi-trait and single-trait models.

Scheme	Trait	Multi-trait				Single-trait			
		Model calibration		Test		Model calibration		Held-out test	
		R^2^	RMSE	R^2^	RMSE	R^2^	RMSE	R^2^	RMSE
BP-ANN	PNmax	0.81	1.70	0.53	3.80	0.90	1.26	0.76	2.75
	Isat	0.70	195.14	0.24	461.64	0.90	111.01	**0.59**	**343.72**
	Icomp	0.74	6.63	**0.67**	**9.61**	0.89	4.33	0.28	16.17
	Rd	0.70	0.35	**0.71**	**0.48**	0.88	0.22	0.17	0.93
CNN	PNmax	0.75	1.94	0.68	3.24	0.88	1.33	**0.78**	**2.69**
	Isat	0.6	223.5	0.28	454.18	0.59	221.1	0.55	368.22
	Icomp	0.78	6.38	0.4	13.49	0.94	3.27	**0.61**	**10.19**
	Rd	0.73	0.34	0.37	0.72	0.71	0.35	0.39	0.68
SVR	PNmax	0.62	2.37	**0.79**	**2.92**	0.85	1.55	0.55	3.68
	Isat	0.36	274.32	**0.48**	**404.2**	0.79	163.03	0.44	399.97
	Icomp	0.61	8.06	0.57	10.88	0.85	5.01	0.33	14.5
	Rd	0.57	0.42	0.5	0.62	0.5	0.45	**0.43**	**0.65**
BP-ANN+CARS	PNmax	0.81	1.68	0.79	2.77	0.95	0.92	0.77	2.87
	Isat	0.59	220.82	**0.64**	**342.27**	0.87	123.65	**0.63**	**374.69**
	Icomp	0.81	5.67	**0.66**	**9.52**	0.81	5.55	0.43	12.67
	Rd	0.76	0.31	0.64	0.53	0.90	0.21	**0.60**	**0.57**
CNN+CARS	PNmax	0.73	2.04	0.76	2.75	0.88	1.33	**0.78**	**2.69**
	Isat	0.57	227.48	0.54	363.74	0.83	148.28	0.45	397.82
	Icomp	0.81	5.84	0.65	9.93	0.94	3.27	**0.61**	**10.19**
	Rd	0.74	0.33	**0.76**	**0.46**	0.86	0.23	0.34	0.73
SVR+CARS	PNmax	0.68	2.18	**0.80**	**2.71**	0.66	2.24	0.72	3.21
	Isat	0.43	258.75	0.65	367.13	0.98	52.81	0.38	433.97
	Icomp	0.64	7.74	0.57	11.00	0.87	4.63	0.49	12.84
	Rd	0.61	0.40	0.58	0.57	0.60	0.40	0.45	0.64

Bold values indicate the best performance on the held-out test set (highest R² and lowest RMSE) for each trait across BP-ANN, CNN, and SVR, evaluated separately for the multi-trait and single-trait frameworks and for models with and without CARS variable selection.

#### 3.4.1 BP-ANN model performance

Overall, for both MT-BP-ANN and ST-BP-ANN models, the calibration accuracies were comparable between models with CARS and full-spectrum. However, notable differences emerged in test. Models with CARS achieved higher predictive accuracy, with R^2^ of 0.64 to 0.79 for MT-BP-ANN and 0.43 to 0.77 for ST-BP-ANN, whereas models with full-spectrum performed substantially worse, with R^2^ values of 0.24 to 0.71 for MT-BP-ANN and 0.17 to 0.76 for ST-BP-ANN. These results demonstrate that although BP-ANN models are capable of fitting the training data even without variable selection, the incorporation of CARS markedly enhances their generalization.

For the BP-ANN+CARS scheme, the ST-BP-ANN models achieved the highest calibration accuracies across all traits, with R^2^ values ranging from 0.81 to 0.95. The MT-BP-ANN models exhibited slightly lower calibration accuracies (R^2^ = 0.59 to 0.81), yet maintained competitive test performance (R^2^ = 0.64 to 0.79) across all traits. In particular, for Icomp, the MT-BP-ANN model outperformed the corresponding ST-BP-ANN model on the test set, achieving a higher R^2^ (0.66 vs. 0.43) and a lower RMSE (9.52 vs. 12.67 μmol (photon) m^−2^ s^−1^).

Overall, while ST-BP-ANN models excel in trait-specific calibration accuracy, MT-BP-ANN models provide a more balanced and generalizable prediction framework, making them advantageous for simultaneous MT estimation in cases with strong trait interdependencies.

The best-performing configuration was the MT-BP-ANN model combined with CARS variable selection, which was trained using the Levenberg–Marquardt (trainlm) algorithm. This network employed a single hidden layer with eight neurons and incorporated a regularization coefficient of 0.2 to prevent overfitting. The predictive performance of the optimal BP-ANN model is presented in the scatter plots (**Figure 6**), where the predicted values of all photosynthetic phenotyping traits are plotted against the corresponding observations across different nitrogen levels (N0–N3) and leaf positions (L1–L3). The close agreement of data points with the 1:1 reference line in the scatter plots highlights the strong predictive ability and robustness of the MT-BP-ANN+CARS scheme under varying physiological and experimental conditions. Notably, MT-BP-ANN achieved higher test accuracies for PNmax (R^2^ = 0.79, RMSE = 2.77 μmol (CO_2_) m^−2^ s^−1^), Isat (R^2^ = 0.64, RMSE = 342.27 μmol (photon) m^−2^ s^−1^), Icomp (R^2^ = 0.66, RMSE = 9.52 μmol (photon) m^−2^ s^−1^), and Rd (R^2^ = 0.64, RMSE = 0.53 μmol (CO_2_) m^−2^ s^−1^) compared with the corresponding ST-BP-ANN models. In comparison, ST-BP-ANN+CARS (**Supplementary Figure S3**) achieved comparable calibration accuracy but showed reduced performance in test, highlighting the benefit of MT-BP-ANN integrating multiple traits within a single model.

**Figure 6:**
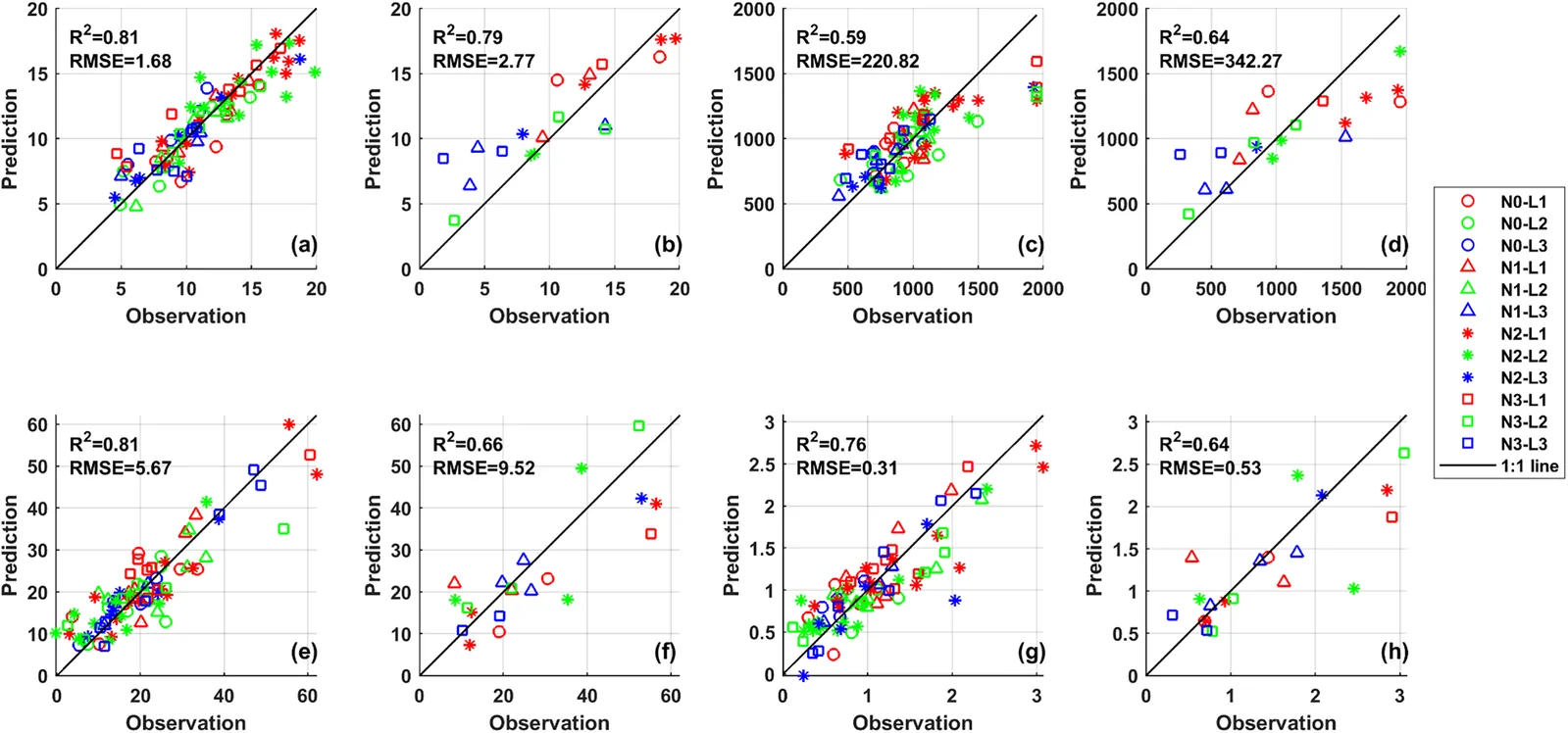
Scatter plots of observed versus predicted values for photosynthetic phenotyping traits (PNmax **(a, b)**, Isat **(c, d)**, Icomp **(e, f)**, and Rd **(g ,h)**) obtained using MT-BP-ANN combined with CARS under different nitrogen levels (N0–N3) and leaf positions (L1–L3). Panels **(a, c, e, g)** show the calibration results, whereas panels **(b, d, f, h)** show the test results. The black 1:1 line represents perfect agreement between observed and predicted values.

#### 3.4.2 CNN model performance

For ST-CNN, model accuracies were comparable between CARS and full-spectrum. CNN models combined with CARS also showed competitive performance (**Table 3**). For MT-CNN, calibration accuracies were similar with CARS and full-spectrum, but test improved markedly when CARS was applied (R^2^ = 0.54 to 0.76 vs. 0.28 to 0.68). These patterns mirror those of the CNN models, where the benefit of CARS was largest in the MT setting, supporting its role in improving MT generalization.

For the CNN+CARS scheme, MT-CNN models exhibited slightly lower calibration accuracy (R^2^ = 0.57 to 0.81) compared with the ST-CNN models, which maintained high calibration performance (R^2^ = 0.83 to 0.94). Despite the slightly lower calibration performance, MT-CNN demonstrated stable test accuracy across all traits (R^2^ = 0.54 to 0.76). Specifically, the test performance for Isat (R^2^ = 0.54, RMSE = 363.74 μmol (photon) m^−2^ s^−1^), Icomp (R^2^ = 0.65, RMSE = 9.93 μmol (photon) m^−2^ s^−1^), and Rd (R^2^ = 0.76, RMSE = 0.46 μmol (CO_2_) m^−2^ s^−1^) showed noticeable improvements compared with the corresponding ST-CNN models.

The best-performing configuration was the MT-CNN model combined with CARS variable selection. Each convolutional block consisted of a one-dimensional convolutional layer (kernel size = 5, 16 filters), followed by batch normalization, a rectified linear unit (ReLU) activation function, and a dropout layer with a rate of 0.2. The network was trained with a learning rate of 0.001 and a batch size of 8. The predictive performance of the optimal CNN model is shown in the scatter plots (**Figure 7**), where predicted and observed values of all traits are compared across nitrogen levels (N0–N3) and leaf positions (L1–L3). The close alignment with the 1:1 line highlights the strong accuracy and robustness of the MT-CNN+CARS model under varying conditions. In comparison, ST-CNN+CARS (**Supplementary Figure S4**) yielded similar calibration performance but lower test accuracy, confirming that the MT-CNN framework provides more reliable generalization by jointly modeling multiple traits within a single network.

**Figure 7:**
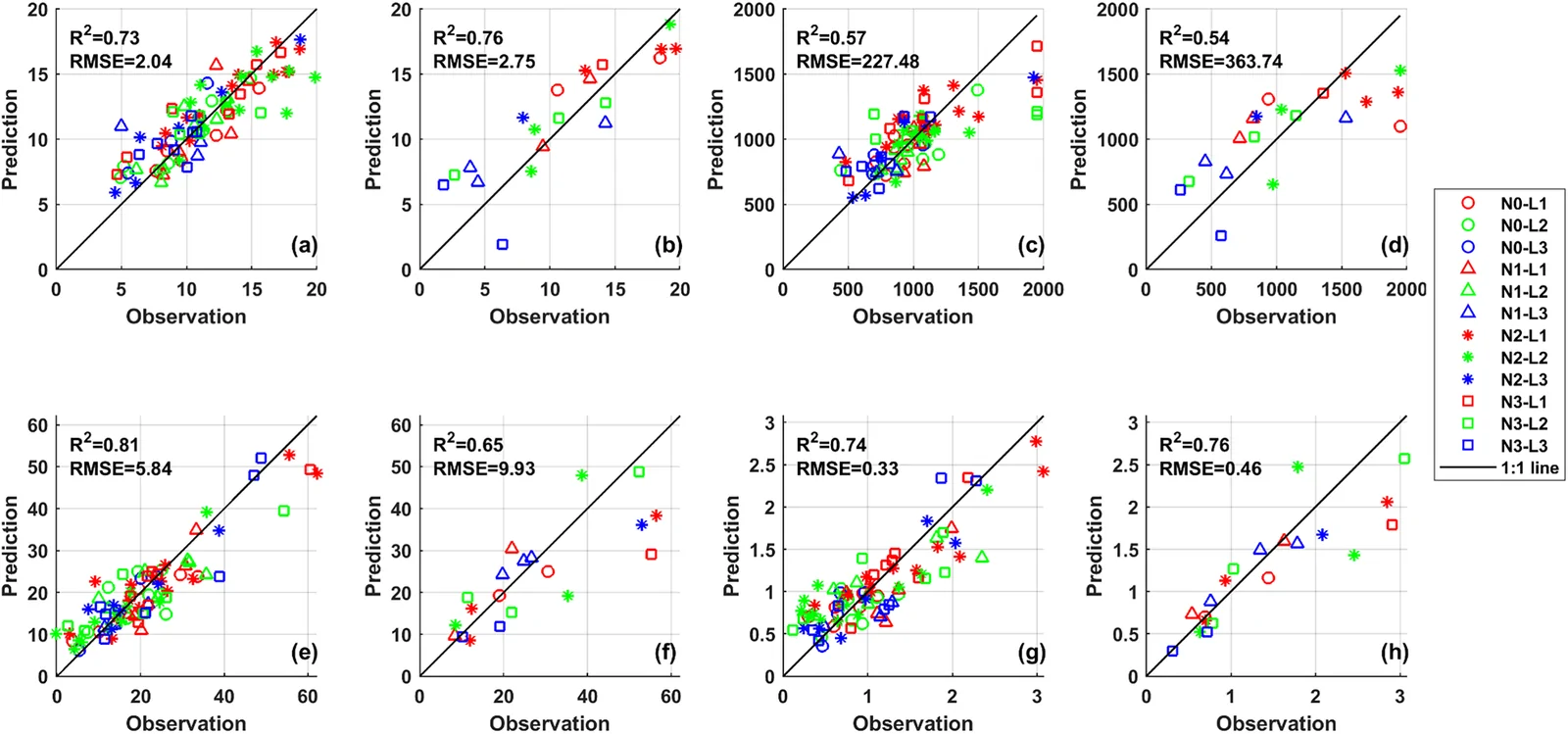
Scatter plots of observed versus predicted values for photosynthetic phenotyping traits (PNmax **(a, b)**, Isat **(c, d)**, Icomp **(e, f)**, and Rd **(g, h)**) obtained using MT-CNN combined with CARS under different nitrogen levels (N0–N3) and leaf positions (L1–L3). Panels **(a, c, e, g)** show the calibration results, whereas panels **(b, d, f, h)** show the test results. The black 1:1 line represents perfect agreement between observed and predicted values.

#### 3.4.3 SVR model performance

SVR models with CARS consistently outperformed those with full-spectrum. For MT-SVR, the test R^2^ with the full spectrum across four traits were 0.48 to 0.79, while with CARS test R^2^ were 0.57 to 0.80.

Coupled with CARS, the MT-SVR models displayed moderately lower calibration (R^2^ = 0.43 to 0.68) than ST-SVR models (calibration R^2^ = 0.60 to 0.98), but the MT-SVR yielded higher test accuracy. The best-performing configuration was the MT-SVR model with CARS variable selection (RBF kernel, kernel width = 10.58, penalty C = 3511.20, and ϵ-insensitive=0.01). The predictive performance is illustrated in the scatter plots (**Supplementary Figure S5**), for PNmax (R^2^ = 0.80, RMSE = 2.71 μmol (CO_2_) m^−2^ s^−1^), Isat (R^2^ = 0.65, RMSE = 367.13 μmol (photon) m^−2^ s^−1^), Icomp (R^2^ = 0.57, RMSE = 11.00 μmol (photon) m^−2^ s^−1^), and Rd (R^2^ = 0.58, RMSE = 0.57 μmol (CO_2_) m^−2^ s^−1^), outperforming the ST-SVR+CARS counterpart (**Supplementary Figure S6**).

## 4 Discussion

The MT framework is advantageous for reasons that go beyond a higher test performance. The four LRC-derived traits, PNmax, Isat, Icomp, and Rd, are not statistically independent variables but coupled descriptors of the leaf-level photosynthetic system, jointly governed by pigment composition, electron transport capacity, and mesophyll structure. A single hyperspectral scan therefore carries information that is simultaneously relevant to all four parameters, and a model that exploits this coupling is mechanistically aligned with the deployment scenario of high-throughput field phenotyping, where the relevant phenotype is the joint physiological state of the leaf rather than any one parameter in isolation (Waegeman et al., 2019). From an operational standpoint, MT also collapses four trait-specific training and tuning workflows into one, removing the asymmetric calibration and maintenance burden of running four separate ST pipelines for every new variety, season, or sensor configuration. Correlation analyses confirm close associations among the four parameters (**Figure 3**). This finding is consistent with previous studies reporting that multivariate approaches such as PLSR2 and MT-CNN are particularly suitable when response variables exhibit strong correlations, as they can effectively capture shared variance and improve prediction accuracy (Blanco and Peguero, 2008; Abdel-Rahman et al., 2017; Zhai et al., 2025). Although ST models yielded relatively high calibration performance, their predictive performance was generally lower on the held-out test set (**Tables 2**, **3**). This consistent MT framework is precisely what enables scaling from per-leaf LI-6400 measurements to breeding-panel screening and field-scale photosynthetic capacity assessment.

Typically, a hyperspectral reflectance spectrum contains a large number of predictor variables, representing reflectance at hundreds to thousands of wavelengths. In the present study, each spectrum contained 451 wavelength variables, whereas the dataset comprised 104 observations. Hyperspectral data are therefore high-dimensional and often strongly collinear (Wold et al., 2001; Heckmann et al., 2017; Burnett et al., 2021). Models constructed with full-spectrum inputs may contain substantially more predictor variables than observations, increasing model complexity and the risk of overfitting (Burnett et al., 2021). Consistent with this concern, the full-spectrum models in the present study generally showed lower predictive performance on the held-out test set than their wavelength-selected counterparts (**Tables 2**, **3**). Variable selection is therefore essential for reducing dimensionality, alleviating bias, and enhancing model robustness. Most conventional variable selection approaches, however, are designed for ST regression problems, where the goal is to optimize prediction for one target variable. In the context of MT estimation, extracting shared informative features that can simultaneously predict multiple correlated photosynthetic phenotyping traits remains under-explored. To address this gap, we extended classical variable selection algorithms—SPA, UVE, and CARS—by integrating them with the PLSR2 framework, enabling the identification of common spectral features that are jointly relevant to PNmax, Isat, Icomp, and Rd. The common spectral features identified across the three methods were predominantly located in the green (533–537 nm, close to wavelengths sensitive to xanthophyll-cycle activity), red (654–662 nm, coinciding with strong chlorophyll absorption), red-edge (711–714 nm, highly sensitive to chlorophyll variation), and NIR regions (813–819 nm, 881–888 nm, 899–904 nm, and 920–935 nm) (**Figure 4**), which are influenced by internal leaf structure and multiple scattering (Jacquemoud and Baret, 1990; Ollinger, 2011). These selected spectral regions overlap with or are close to wavelengths used in established VIs related to photosynthetic function, such as the PRI (Gamon et al., 1992), CI_red-edge_ (Gitelson et al., 2003), the CCI (Gamon et al., 2016), and the near-infrared reflectance of vegetation (NIRv) (Badgley et al., 2017). Consequently, these spectral regions may contain complementary information related to variation in PNmax, Isat, Icomp, and Rd, although the four LRC-derived traits are not expected to have identical spectral responses. Variable selection substantially enhanced model performance, particularly on the held-out test set. For example, PLSR2 + CARS improved the held-out test-set R^2^ to 0.52–0.84 across the four traits, compared with 0.46–0.81 using the full-spectrum (**Table 2**). In addition to enhancing prediction accuracy, selecting common informative bands offers practical value for the development of compact, portable instruments, such as the PlantPen PRI 210 (Tsoumalakou et al., 2023), PlantPen NDVI 310 (Orzechowska et al., 2026), GreenSeeker Handheld Crop Sensor (Kitić et al., 2019) and RedEdge-P sensor (Ali et al., 2023). These findings provide candidate wavebands for the future development of compact spectral sensors capable of simultaneously monitoring multiple photosynthetic phenotyping traits, although further validation using held-out datasets and purpose-built instruments is required.

Comparative studies of PLSR and machine learning models in hyperspectral phenotyping have reported varying results across plant species, sensor characteristics, experimental designs, and environmental conditions (Heckmann et al., 2017). PLSR provides a strong baseline for spectral regression because it projects numerous collinear wavelength variables onto a relatively small number of latent variables that capture the covariance structure between spectral predictors and response variables, resulting in a parsimonious framework that can perform effectively with small to moderate calibration datasets (Fu et al., 2019; Burnett et al., 2021). PLSR primarily models linear relationships between spectral predictors and target traits, whereas machine learning approaches can accommodate more complex and nonlinear spectral–trait relationships. However, the greater flexibility of nonlinear machine learning models, particularly neural networks, can increase their sensitivity to sample size, model architecture, regularization, and hyperparameter tuning and may lead to a greater risk of overfitting when calibration data are limited. Accordingly, previous comparisons have variously favored PLSR, nonlinear machine learning models, or ensemble approaches depending on the target trait, plant material, spectral preprocessing, feature-selection strategy, and validation procedure (Heckmann et al., 2017; Fu et al., 2019; Wijewardane et al., 2023). Our results similarly did not identify a universally superior model. With CARS wavelength selection, PLSR2 achieved the highest test R^2^ for PNmax (0.84) and Isat (0.69), whereas MT-BP-ANN achieved the highest test R^2^ for Icomp (0.66), and MT-CNN achieved the highest test R^2^ for Rd (0.76) (**Tables 2**, **3**). These results indicate that model performance was trait-dependent: the relatively parsimonious PLSR2 framework was particularly effective for PNmax and Isat, whereas nonlinear multi-trait models showed better predictive performance for Icomp and Rd in the present dataset.

A total of 104 LRCs were collected across three years, three rice varieties, four nitrogen levels, three irrigation regimes, different leaf positions, and multiple measurement dates, providing substantial physiological variability for the development and evaluation of the PLSR and machine learning models. However, these experimental factors were neither fully crossed nor equally represented. Variety was partially confounded with year, because Huayou14 was measured in 2015–2016, whereas Huhan61 was used in the 2017 nitrogen experiment and Xiushui134 was included only in the 2017 irrigation experiment. In addition, the irrigation experiment contained only 12 observations, was conducted at N2, and included only the L1 and L2 leaf positions. Therefore, the observed variation among observations should be interpreted as the combined effect of genotype, management, leaf position, developmental status, and environmental conditions rather than as independent effects of individual experimental factors.

The dataset was divided into training and held-out test sets using stratified random sampling according to the distributions of PNmax, Isat, Icomp, and Rd, rather than by year, variety, or treatment. As shown in **Figures 5**-**7**, observations from different experimental conditions were distributed across a broad range of measured and predicted LRC related traits, indicating that the spectral models captured variation in photosynthetic traits occurring under heterogeneous field conditions. Nevertheless, because observations from the same varieties, years, and treatments could occur in both the training and test sets, the present results should not be interpreted as independent test for unseen varieties, years, or management treatments. Future studies using larger and more balanced datasets with factorial experimental designs and leave-one-variety-, treatment-, or year-out validation will be required to quantify the independent effects of these factors and more rigorously assess model transferability across growing conditions.

Building on these findings, MT hyperspectral phenotyping could be advanced along three directions. First, expanding datasets to include more diverse rice varieties, environmental conditions, growth stages, and stress treatments would enhance model generalization and robustness, particularly for the machine learning models. Second, integrating complementary sensing modalities, such as thermal imaging and chlorophyll fluorescence, may provide additional physiological information and enable simultaneous monitoring of more complex traits. Third, developing more efficient MT variable selection and modeling frameworks that can handle larger, heterogeneous datasets will be essential for real-time deployment in field-scale applications.

## 5 Conclusions

The integration of MT variable selection and MT regression provides a robust framework for capturing the complex physiological relationships among photosynthetic traits. This study demonstrated that variable selection substantially enhances model performance, particularly in model test. PLSR2, MT-BP-ANN, MT-CNN, and MT-SVR models can simultaneously estimate four key photosynthetic phenotyping traits (PNmax, Isat, Icomp, and Rd). Overall, the linear PLSR2+CARS model provided the best balance of predictive accuracy, robustness, and model simplicity across the four traits. PLSR2+CARS delivered the highest test accuracy for PNmax and Isat, MT-BP-ANN+CARS delivered the highest test accuracy for Icomp, and MT-CNN+CARS delivered the highest test accuracy for Rd. These findings highlight the potential of this approach for nondestructive, high-throughput field phenotyping, providing a field-deployable tool for crop breeding and precision crop management. By compressing per-leaf LRC-trait acquisition from approximately 60 minutes (LI-6400) to a single hyperspectral scan within 1 minute, the framework removes a key bottleneck for field-scale leaf photosynthetic phenotyping and enables high-throughput screening in breeding trials and stress-response monitoring.

## Data Availability

The raw data supporting the conclusions of this article will be made available by the authors, without undue reservation.
