# Supplementary material for: Hyperspectral multi-trait modeling for rapid assessment of rice photosynthetic light-response traits under field conditions
Source: Front Plant Sci. 2026 Oct 13;17:1968739. doi: 10.3389/fpls.2026.1968739 (PMC13710402; doi:10.3389/fpls.2026.1968739)
Supplement: Supplementary file 1 [file DataSheet1.docx]

Supplementary Material

# 1 Supplementary Figures and Tables

## 1.1 Supplementary Table

Supplementary Table 1: Experimental characteristics and training/test assignment of the 104 leaf-level observations used in this study

| **Sample No.** | **Dataset** | **Label** | **Cultivar** | **Sample No.** | **Dataset** | **Label** | **Cultivar** |
| --- | --- | --- | --- | --- | --- | --- | --- |
| 1 | Training | 20150909-N2-L1 | Huayou14 | 28 | Training | 20160912-N2-L3 | Huayou14 |
| 2 | Training | 20150909-N2-L2 | Huayou14 | 29 | Test | 20160912-N3-L2 | Huayou14 |
| 3 | Training | 20150909-N2-L3 | Huayou14 | 30 | Test | 20160920-N0-L1 | Huayou14 |
| 4 | Training | 20150909-N3-L1 | Huayou14 | 31 | Training | 20160920-N0-L2 | Huayou14 |
| 5 | Training | 20150909-N3-L2 | Huayou14 | 32 | Training | 20160920-N0-L3 | Huayou14 |
| 6 | Training | 20150909-N3-L3 | Huayou14 | 33 | Training | 20160920-N1-L1 | Huayou14 |
| 7 | Training | 20151002-N0-L1 | Huayou14 | 34 | Training | 20160920-N1-L2 | Huayou14 |
| 8 | Training | 20151002-N0-L2 | Huayou14 | 35 | Training | 20160920-N1-L3 | Huayou14 |
| 9 | Training | 20151002-N0-L1 | Huayou14 | 36 | Training | 20160925-N2-L1 | Huayou14 |
| 10 | Training | 20151002-N0-L2 | Huayou14 | 37 | Training | 20160925-N2-L2 | Huayou14 |
| 11 | Training | 20151002-N1-L1 | Huayou14 | 38 | Training | 20160925-N2-L3 | Huayou14 |
| 12 | Training | 20151002-N1-L2 | Huayou14 | 39 | Training | 20160925-N3-L1 | Huayou14 |
| 13 | Training | 20151002-N2-L1 | Huayou14 | 40 | Training | 20160925-N3-L2 | Huayou14 |
| 14 | Training | 20151002-N2-L2 | Huayou14 | 41 | Training | 20160925-N3-L3 | Huayou14 |
| 15 | Training | 20151002-N2-L3 | Huayou14 | 42 | Training | 20161024-N0-L1 | Huayou14 |
| 16 | Training | 20151002-N3-L1 | Huayou14 | 43 | Training | 20161024-N0-L2 | Huayou14 |
| 17 | Training | 20151002-N3-L2 | Huayou14 | 44 | Training | 20161024-N1-L1 | Huayou14 |
| 18 | Test | 20151002-N3-L3 | Huayou14 | 45 | Training | 20161024-N1-L2 | Huayou14 |
| 19 | Training | 20151002-N2-L1 | Huayou14 | 46 | Test | 20161024-N1-L3 | Huayou14 |
| 20 | Training | 20160912-N0-L1 | Huayou14 | 47 | Training | 20161103-N3-L1 | Huayou14 |
| 21 | Training | 20160912-N0-L2 | Huayou14 | 48 | Test | 20161103-N3-L2 | Huayou14 |
| 22 | Training | 20160912-N0-L3 | Huayou14 | 49 | Test | 20161103-N3-L3 | Huayou14 |
| 23 | Test | 20160912-N1-L1 | Huayou14 | 50 | Test | 20170823-N0-L1 | Huhan61 |
| 24 | Training | 20160912-N1-L2 | Huayou14 | 51 | Training | 20170823-N0-L2 | Huhan61 |
| 25 | Training | 20160912-N1-L3 | Huayou14 | 52 | Training | 20170823-N1-L1 | Huhan61 |
| 26 | Training | 20160912-N2-L1 | Huayou14 | 53 | Training | 20170823-N1-L2 | Huhan61 |
| 27 | Training | 20160912-N2-L2 | Huayou14 | 54 | Test | 20170823-N1-L3 | Huhan61 |
| **Sample No.** | **Dataset** | **Label** | **Cultivar** | **Sample No.** | **Dataset** | **Label** | **Cultivar** |
| 55 | Training | 20170823-N2-L1 | Huhan61 | 80 | Training | 20170913-N3-L3 | Huhan61 |
| 56 | Training | 20170823-N2-L2 | Huhan61 | 81 | Training | 20171007-N0-L1 | Huhan61 |
| 57 | Test | 20170823-N2-L3 | Huhan61 | 82 | Training | 20171007-N0-L2 | Huhan61 |
| 58 | Test | 20170823-N3-L1 | Huhan61 | 83 | Training | 20171007-N0-L3 | Huhan61 |
| 59 | Test | 20170823-N3-L2 | Huhan61 | 84 | Test | 20171007-N1-L1 | Huhan61 |
| 60 | Training | 20170823-N3-L3 | Huhan61 | 85 | Training | 20171007-N1-L2 | Huhan61 |
| 61 | Training | 20170828-N2-L1 | Huhan61 | 86 | Test | 20171007-N1-L3 | Huhan61 |
| 62 | Test | 20170828-N2-L2 | Huhan61 | 87 | Training | 20171007-N2-L1 | Huhan61 |
| 63 | Training | 20170828-N2-L3 | Huhan61 | 88 | Test | 20171007-N2-L2 | Huhan61 |
| 64 | Training | 20170828-N3-L1 | Huhan61 | 89 | Training | 20171007-N2-L3 | Huhan61 |
| 65 | Training | 20170828-N3-L2 | Huhan61 | 90 | Training | 20171007-N3-L1 | Huhan61 |
| 66 | Training | 20170828-N3-L3 | Huhan61 | 91 | Training | 20171007-N3-L2 | Huhan61 |
| 67 | Test | 20170828-N1-L1 | Huhan61 | 92 | Training | 20171007-N3-L3 | Huhan61 |
| 68 | Test | 20170828-N1-L2 | Huhan61 | 93 | Training | 20170912-HH20-L1 | Huhan61 |
| 69 | Training | 20170913-N0-L1 | Huhan61 | 94 | Training | 20170912-HH20-L2 | Huhan61 |
| 70 | Training | 20170913-N0-L2 | Huhan61 | 95 | Test | 20170912-HH50-L1 | Huhan61 |
| 71 | Training | 20170913-N0-L3 | Huhan61 | 96 | Training | 20170912-HH50-L2 | Huhan61 |
| 72 | Training | 20170913-N1-L1 | Huhan61 | 97 | Training | 20170912-HH100-L1 | Huhan61 |
| 73 | Training | 20170913-N1-L2 | Huhan61 | 98 | Training | 20170912-HH100-L2 | Huhan61 |
| 74 | Training | 20170913-N1-L3 | Huhan61 | 99 | Test | 20170912-XS20-L1 | Xiushui134 |
| 75 | Training | 20170913-N2-L1 | Huhan61 | 100 | Training | 20170912-XS20-L2 | Xiushui134 |
| 76 | Training | 20170913-N2-L2 | Huhan61 | 101 | Training | 20170912-XS50-L1 | Xiushui134 |
| 77 | Training | 20170913-N2-L3 | Huhan61 | 102 | Training | 20170912-XS50-L2 | Xiushui134 |
| 78 | Training | 20170913-N3-L1 | Huhan61 | 103 | Training | 20170912-XS100-L1 | Xiushui134 |
| 79 | Training | 20170913-N3-L2 | Huhan61 | 104 | Training | 20170912-XS100-L2 | Xiushui134 |

Note: Labels encode the measurement date and experimental information. For the nitrogen experiment, for example, 20170913-N3-L2 denotes a sample measured on 13 September 2017 under the N3 nitrogen treatment at leaf position L2. For the irrigation experiment, the label additionally includes an abbreviation of the cultivar; for example, 20170912-HH20-L1 denotes Huhan61 (HH) under the 20% irrigation treatment, whereas 20170912-XS50-L1 denotes Xiushui134 (XS) under the 50% irrigation treatment.

## 1.2 Supplementary Figures

**Supplementary Figure 1.** Wavelengths selected by three variable selection methods (CARS, SPA, and UVE) for the prediction of individual photosynthetic phenotyping traits: PNmax (a), Isat (b), Icomp (c), and Rd (d). The black line represents the mean reflectance spectrum of all samples, while the colored markers indicate the positions of the wavelengths selected by each algorithm.

**Supplementary Figure 2.** Scatter plots of observed versus predicted values for photosynthetic phenotyping traits (PNmax (a, b), Isat (c, d), Icomp (e, f), and Rd (g, h)) obtained using PLSR1 combined with CARS under different nitrogen levels (N0–N3) and leaf positions (L1–L3). Panels a, c, e, and g show the calibration results, whereas panels b, d, f, and h show the test results. The black 1:1 line represents perfect agreement between observed and predicted values.

**Supplementary Figure 3.** Scatter plots of observed versus predicted values for photosynthetic phenotyping traits (PNmax (a, b), Isat (c, d), Icomp (e, f), and Rd (g, h)) obtained using ST-BP-ANN combined with CARS under different nitrogen levels (N0–N3) and leaf positions (L1–L3). Panels a, c, e, and g show the calibration results, whereas panels b, d, f, and h show the test results. The black 1:1 line represents perfect agreement between observed and predicted values.

**Supplementary Figure 4.** Scatter plots of observed versus predicted values for photosynthetic phenotyping traits (PNmax (a, b), Isat (c, d), Icomp (e, f), and Rd (g, h)) obtained using ST-CNN combined with CARS under different nitrogen levels (N0–N3) and leaf positions (L1–L3). Panels a, c, e, and g show the calibration results, whereas panels b, d, f, and h show the test results. The black 1:1 line represents perfect agreement between observed and predicted values.

**Supplementary Figure 5.** Scatter plots of observed versus predicted values for photosynthetic phenotyping traits (PNmax (a, b), Isat (c, d), Icomp (e, f), and Rd (g, h)) obtained using MT-SVR combined with CARS under different nitrogen levels (N0–N3) and leaf positions (L1–L3). Panels a, c, e, and g show the calibration results, whereas panels b, d, f, and h show the test results. The black 1:1 line represents perfect agreement between observed and predicted values.

**Supplementary Figure 6.** Scatter plots of observed versus predicted values for photosynthetic phenotyping traits (PNmax (a, b), Isat (c, d), Icomp (e, f), and Rd (g, h)) obtained using ST-SVR combined with CARS under different nitrogen levels (N0–N3) and leaf positions (L1–L3). Panels a, c, e, and g show the calibration results, whereas panels b, d, f, and h show the test results. The black 1:1 line represents perfect agreement between observed and predicted values.
